# The Role of Alternative RNA Splicing in the Regulation of *hTERT*, Telomerase, and Telomeres: Implications for Cancer Therapeutics

**DOI:** 10.3390/cancers12061514

**Published:** 2020-06-10

**Authors:** Aaron L. Slusher, Jeongjin JJ Kim, Andrew T. Ludlow

**Affiliations:** School of Kinesiology, University of Michigan, Ann Arbor, MI 48109, USA; alslush@umich.edu (A.L.S.); snujjk@umich.edu (J.J.K.)

**Keywords:** alternative RNA Splicing, oligonucleotide therapeutics, telomeres, telomerase, *hTERT*

## Abstract

Alternative RNA splicing impacts the majority (>90%) of eukaryotic multi-exon genes, expanding the coding capacity and regulating the abundance of gene isoforms. Telomerase (*hTERT*) is a key example of a gene that is alternatively spliced during human fetal development and becomes dysregulated in nearly all cancers. Approximately 90% of human tumors use telomerase to synthesize *de novo* telomere repeats and obtain telomere-dependent cellular immortality. Paradigm shifting data indicates that *hTERT* alternative splicing, in addition to transcription, plays an important role in the regulation of active telomerase in cells. Our group and others are pursuing the basic science studies to progress this emerging area of telomerase biology. Recent evidence demonstrates that switching splicing of *hTERT* from the telomerase activity producing full-length *hTERT* isoform to alternatively spliced, non-coding isoforms may be a novel telomerase inhibition strategy to prevent cancer growth and survival. Thus, the goals of this review are to detail the general roles of telomerase in cancer development, explore the emerging regulatory mechanisms of alternative RNA splicing of the *hTERT* gene in various somatic and cancer cell types, define the known and potential roles of *hTERT* splice isoforms in cancer cell biology, and provide insight into new treatment strategies targeting *hTERT* in telomerase-positive cancers.

## 1. Introduction

Nearly 40% of adults in the United States are expected to be diagnosed with cancer in their lifespan [1]. In 2020 alone, it is estimated that cancer will account for over 600,000 deaths, making cancer the second leading cause of mortality behind cardiovascular disease [2]. Incidence, prevalence, and mortality rates vary widely among various types of cancers, and the search for a central mechanism remains at the forefront of cancer therapeutic discovery efforts [1]. Cellular immortality is considered a requisite for the generation of macroscopic tumors. The acquisition of cellular immortality is conferred by the maintenance of telomeres during many cellular divisions that sustain cancer cell growth and survival [3]. Therefore, research into cancer therapeutics targeting telomeres and associated regulatory mechanisms have been the subject of intense study for nearly 30 years [4].

Human telomeres are tandem repeats of 5′-TTAGGG_n_ DNA sequences located at the ends of linear chromosomes [5]. The telomere protects the linear chromosomal ends from end-to-end fusion and being recognized as damaged DNA [6,7,8,9]. During DNA replication, telomeres lose a small segment of DNA (30–150 nt) and progressively shorten in length with each cycle of cellular replication due to the inability of DNA polymerase to completely replicate the lagging strand of telomeric DNA [10,11,12]. The “end replication problem” was hypothesized by Watson, and separately Olovnikov, to require specialized mechanisms to maintain telomeres [13,14]. Indeed, Greider and Blackburn discovered that the telomerase enzyme partially compensates for telomere attrition in a ciliate (*Tetrahymena thermophila*) [15]. Shortly thereafter, the telomerase enzyme was discovered in humans as a ribonucleoprotein (RNP) that is assembled and recruited to the ends of telomeres to synthesize de novo TTAGGG repeats necessary for their maintenance and potential elongation at the ends of chromosomes [16]. During development, however, telomerase enzyme activity is suppressed in normal somatic cells [17,18]. As a result, telomere lengths shorten progressively with age and continued replication of cellular DNA, and upon reaching a critically shortened length, cells enter a state of growth arrest referred to as replicative senescence. This senescence stage (M1) signals the replicative capacity for cellular division, termed the “Hayflick limit” [19], and serves as a barrier to prevent cells from progressing to malignant cancer cells in large, long-live mammals, including humans [20,21,22]. However, some cells escape senescence and enter into a crisis state (M2) [23]. During crisis, cells have very short telomeres, their chromosomal ends fuse, and they exhibit genomic instability, whereby cells undergo cellular death by apoptosis [23,24]. Moreover, only 1 in 1·10^7^ human cells are thought to escape crisis and acquire the ability to maintain their very short telomeres necessary to achieve immortality that is characteristic of cancer [25]. This indicates that telomere shortening and replicative senescence are potent blocks to cellular immortality and cancer progression.

Two mechanisms to date have been identified to maintain telomeres in cancer cells. About 10% of tumors use a recombination-based mechanism called alternative lengthening of telomeres (ALT), whereas the majority of tumors (~90%) achieve immortality by reactivating telomerase to maintain their shortened telomere lengths [17,26,27]. Likewise, telomerase inhibition has been shown to contribute to crisis in cancer cells [28]. The telomerase holoenzyme consists of numerous proteins [29,30,31]. The two primary components necessary for telomerase activity include an RNA template (*hTERC*) and a protein catalytic subunit termed telomerase reverse transcriptase (*hTERT*) [29,30,31]. *hTERC* is expressed in somatic cells and telomerase-positive and negative cancers, suggesting that *hTERC* is not typically rate-limiting for the telomerase enzyme nor a good predictor of assessing telomerase activity [32,33]. On the contrary, *hTERT* is tightly regulated and positively correlated with telomerase activity in somatic and cancer cells [34,35,36,37]. More specifically, *hTERT* mRNA expression is absent in telomerase-negative somatic cells, whereas the transient expression of *hTERT* significantly increases telomerase activity [35]. Overexpression of *hTERT* cDNA in telomerase negative cells has further been shown to prevent cellular senescence, increase telomerase activity, and stimulate the lengthening of telomeres in BJ fibroblasts [38,39]. These findings strongly support the notion that *hTERT* is the rate-limiting, catalytic subunit necessary for telomerase enzyme activity, and that *hTERT* alone is sufficient to reconstitute cellular replication and immortality through telomerase activity and telomere length maintenance in most cells. 

As such, *hTERT* has emerged as a potential cancer-related target to attenuate the growth and survival of cancer cells via the inactivation of the telomerase enzyme, the shortening of telomeres, and the induction of cell death by apoptosis. To date, the majority of therapeutic strategies targeting *hTERT* gene regulation in cancer have focused on immunogenic strategies (e.g., vaccination against hTERT antigens) [40,41], targeting somatic mutations within the TERT promoter (−146 C > T; −124 C > T) [42,43], G-quadruplexes [44], and epigenetic modifications [45,46]. Less studied are the roles that alternative splicing play as a post-transcriptional mechanism to regulate *hTERT* gene expression. Therefore, this review will detail the emerging regulatory mechanisms of *hTERT* alternative RNA splicing and processing in various cell types, define the known and potential roles of *hTERT* splice isoforms in cancer cell biology, and provide mechanistic insights that may lead to new treatment strategies targeting the *hTERT* gene in telomerase positive cancers.

## 2. Alternative RNA Splicing as a Mechanism to Regulate Telomerase (*hTERT)*

### 2.1. Alternative RNA Splicing

Splicing of precursor messenger RNA (pre-mRNA) is a conserved biological process that occurs in close relation to RNA polymerase II (Pol II)-mediated transcription in mammals [47]. Pre-mRNA splicing is initiated by the recruitment of the spliceosome, a large RNP complex comprised of five small nuclear RNP (snRNP) particle core components—U1, U2, U4, U5, and U6—and numerous additional proteins [48]. During normal splicing events, the step-wise assembly of the spliceosome components are directed by conserved 5′ and 3′ splice sites (SS) that define exon–intron and intron–exon junctions, respectively, branch point sequences that are located 18–40 nucleotides upstream of the 3′ SS and help to chemically facilitate splicing, and polypyrimidine tracts that proceed and aid in 3′ SS recognition [48,49]. The tightly regulated and coordinated actions of the spliceosome serves to catalyze the formation and subsequent removal of intron lariats from nascent RNA and the ligation of adjacent exons necessary for the production of mature mRNA transcripts containing all available exons in sequential order [48,49]. 

Alternative splicing is a post-transcriptional regulatory mechanism that results in the generation of two or more mRNAs from the same pre-mRNA and is known to impact 92–95% of human multi-exon genes [50,51]. As an evolutionarily conserved RNA metabolism and processing mechanism, alternative splicing, along with intron number, is correlated with organism complexity and helps to explain the proteome diversity identified by the production of over 100,000 proteins from only ~20,000 protein-coding genes observed in humans [52,53,54]. In contrast to constitutive splicing, alternative splicing patterns are represented by cassette exon skipping, intron retention, alternative 5′ and 3′ SS selection within exons, and mutually exclusive alternative exons [55]. Alternative RNA splicing is primarily regulated by *cis*-elements and *trans*-acting RNA binding proteins. More specifically, exonic splicing enhancers (ESEs) and intronic splicing enhancers (ISEs) are *cis*-elements typically bound by *trans*-acting factors, such as members of the serine/arginine-rich splicing factor (SRSF) family of nuclear phosphoproteins that generally promote exon inclusion [56]. Likewise, exonic splicing silencers (ESSs) and intronic splicing silencers (ISSs) are *cis*-elements typically bound by *trans*-acting factors, such as heterogeneous nuclear ribonucleoproteins (hnRNPs) that generally promote exon exclusion or skipping [56]. To date, at least 1542 known *trans*-acting RNA binding proteins have been identified [57], and the combination of the factors within a cell and their interactions with *cis*-elements comprise the “splicing code” that determines the alternative splicing outcome of a particular RNA transcript.

### 2.2. Alternative RNA Splicing of hTERT

*hTERT* is a 42-kb long gene containing 16 exons and 15 introns located on chromosome 5p15.33 [35]. Splicing of *hTERT* pre-mRNA yields the complete 16 exon containing transcript (termed full length [FL]) that contains four specific domains necessary for producing the catalytically active telomerase enzyme: the telomerase N-terminal (TEN)-domain in exon 1, the telomerase RNA-binding domain (TRBD) spanning exons 2 and 3, a reverse transcriptase (RT)-domain spanning exons 4 through 13, and a c-terminal extension (CTE)-domain spanning exons 14 through 16. The RT-domain specifically provides the active sites necessary to catalyze telomerase enzyme activity, is involved in the proper assembly and stabilization of the *hTERT*/*hTERC* complex, and helps to facilitate telomerase enzyme processivity [58,59,60]. Expression of the *hTERT* gene is observed in telomerase-positive cells, including germline cells, embryonic and induced pluripotent stem cells, and in transit amplifying adult progenitor cells, but is thought to be silenced in fully differentiated somatic cells and other mature cell populations (i.e., T cell lymphocytes, quiescent adult stem cells, etc.) that are telomerase negative or express very low levels of the active telomerase enzyme [33,61]. Similarly, the *hTERT* gene is expressed in telomerase-positive immortal cell lines, yet remains absent or alternatively spliced to non-catalytic isoforms in telomerase-negative cancer cells that rely on ALT mechanisms [33,61]. 

The historical paradigm in the telomere/telomerase field was that *hTERT* was transcriptionally silenced during the cellular differentiation process in somatic cells. Shortly after the discovery of *hTERT* in 1997, Meyerson et al. hypothesized that *hTERT* might also be constitutively expressed and subject to post-transcriptional modifications, such as alternative splicing [34]. Within the year, Kilian et al. used RNA from immortal telomerase positive cells and reverse transcription followed by PCR (RT-PCR) with primers that span a section of the RT-domain from exon 5 through 9 that simultaneously detected multiple *hTERT* variants (bands). The longest variant detected contains exons 5–9 and is assumed to be the potentially FL, catalytically active *hTERT* transcript containing all 16 exons required for telomerase enzyme activity. In addition, three alternatively spliced *hTERT* variants were detected that are incapable of producing active telomerase [62]. The three primary alternatively spliced variants detected by the exons 5–9 RT-PCR assay of the *hTERT* gene include: (1) minus alpha (-α)—a deletion of the first 36 nucleotides due to an in-frame cryptic splice site at the 5′ end of exon 6 that serves as a dominant-negative inhibitor of telomerase, resulting in the progressive shortening of telomeres when overexpressed in cells, and subsequently, cellular senescence or apoptotic death [36,63]; (2) minus beta (-β)—the skipping of exons 7 and 8 that is thought to result in a premature stop codon within exon 10, thus targeting the transcript for nonsense-mediated decay [37]; and (3) the -α-β—a variant combining both deletions with a function that should result in degradation by nonsense-mediated decay [36,64]. 

Ulaner et al., using the same exon 5–9 RT-PCR assay as Kilian et al. [62], qualitatively measured *hTERT* splice variants to demonstrate whether or not the *hTERT* gene is transcriptionally repressed during development or undergoes alternative splicing as a mechanism to tightly regulate telomerase activity [65]. Measuring telomerase activity, *hTERC*, and *hTERT* in the fetal liver, heart, and kidney at various stages of human fetal development, Ulaner and colleagues observed three distinct tissue-specific expression patterns. Although *hTERC* gene expression was observed at near constant levels in each tissue during development, expression patterns of the FL and alternatively spliced *hTERT* variants changed throughout development in each tissue. Likewise, telomerase activity tightly correlated with potential FL (exons 5–6–7–8–9 containing) *hTERT* variant expression [65]. More specifically, telomerase activity in the kidney was stable at weeks 8 and 11, progressively decreased at weeks 13 and 15, and was completely absent from weeks 17 through 21. Similarly, the kidney expressed the potential FL, -α, and -β variants at week 8, the potential FL and -β variants from weeks 11 through 17, and only the -β variant from week 18 through 21. This significant shift in *hTERT* splicing away from the potential FL (+α+β), catalytically active variant towards alternatively spliced (-α and/or -β), catalytically inactive variants that lack portions of the RT-domain occurs differently across tissues and at various stages of cellular development. These findings further corroborate the emergent hypothesis that the *hTERT* gene is regulated post-transcriptionally, and that alternative RNA splicing of the *hTERT* gene serves as a mechanism to tightly control telomerase enzyme activity and regulate telomere length [65,66].

Subsequent studies used the primers spanning exons 5–9 of *hTERT* in somatic cells and fibroblast cell lines and did not observe evidence of *hTERT* expression, further supporting the idea that *hTERT* was transcriptionally silenced. However, Hrdlicková et al. used primers in exons located at the 5′ and 3′ end of the *hTERT* gene and identified several additional alternatively spliced variants in telomerase-negative fibroblasts and adult tissues [67]. Additionally, Kim and Ludlow et al. demonstrated that in vitro aged fibroblasts increase *hTERT* expression levels similarly to those observed in cancer cells, but the resulting transcripts never contained critical exons required for the reverse transcriptase activity of telomerase [68]. As such, no telomerase activity was detected. These data have contributed to a new working hypothesis that cells do not completely silence the *hTERT* gene as this would be an energetically wasteful process since stem cells and dividing cells may need small amounts of telomerase to slow telomere shortening during proliferation needed for tissue repair. Instead, somatic cells transcribe a low level of *hTERT* (one to 10 RNA transcripts per cell) and alternatively splice it to non-catalytically active forms. Additionally, since cells only need to maintain the shortest telomeres in a cell to continue to proliferate, only a few molecules of active telomerase are needed to perform this task, and FL *hTERT* can be kept at extremely low levels. Building upon this idea, a paradigm shifting idea has emerged to suggest that when cells transform and progress to a cancerous state they also may not need to increase transcription as long as *hTERT* is spliced to the FL isoform and can generate enough active telomerase to maintain the shortest telomeres in the dividing portion of tumor cells. Indeed, our laboratory has shown that cancer cells use a post-transcriptionally based mechanism to generate telomerase activity from very low levels of *hTERT* transcripts. These landmark studies paved the way for a new line of thinking that *hTERT* alternative RNA splicing can be targeted as a potential cancer therapeutic. Understanding the regulation of *hTERT* alternative RNA splicing is necessary to develop a new and more potent telomerase inhibitor to stop the long-term proliferation of malignant cells.

### 2.3. Alternative RNA Splicing of hTERT in Cancer

Cancer is characterized by aberrant regulation of alternative splicing [69]. Changes in splicing during cancer may result from numerous factors, including mutations to *cis* regulatory sequences or *trans*-acting splicing proteins, modifications to Pol II transcriptional rates, and changes in the expression of and/or post-transcriptional modifications of *trans*-acting splicing proteins [70]. As a result, shifts in splicing are involved in the promotion of characteristics contributing to tumorigenesis, such as an epithelial-to-mesenchymal transition, advanced cellular proliferation, and resistance to apoptosis [71]. Due to the tight regulation of the telomerase enzyme during cellular differentiation and maturation, and the re-emergence of telomerase during cancer, alternative RNA splicing of *hTERT* is likely subjected to either dysregulation or the re-emergence of a developmentally regulated splicing program to maintain telomere lengths during cancer development. 

Kilian et al. first demonstrated that cancer cells display an incredible diversity of alternatively spliced *hTERT* variants [62]. Including FL, 22 variants of the *hTERT* gene have thus far been identified [67]. While the research is ongoing to fully elucidate functions of each variant, alternatively spliced variants within the RT domain of exons 5–9 remain the most widely studied due to the presence of exon 7 and 8 skipping within the RT domain (-β variant). In fact, subsequent studies have quantified that the FL and -β *hTERT* variants combine for >90% of the steady state transcripts observed in cancer cells, whereas the -α and -α-β variants typically account for <5% of the remaining steady state transcripts [37,72,73]. 

**Figure 1:**
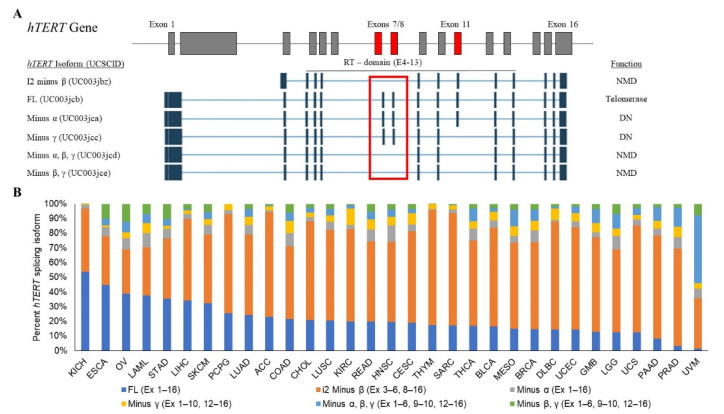
Common *hTERT* gene isoforms and prevalence observed across 31 tumor types as determined by the TSVdb. (**A**) A cartoon of the common *hTERT* splicing isoform structures observed across 31 tumors. Only the complete 16 exon containing FL transcript produces the catalytically active telomerase enzyme. On the contrary, all other alternatively spliced, catalytically inactive isoforms that include a combination of the -α, -β, and -γ exon skipping events are known or predicted to act as a dominant negative or target the transcript for nonsense-mediated decay, and thus yield cells telomerase negative. (**B**) The percentage of each *hTERT* splicing isoform categorized in 31 individual tumors types, demonstrating that the -β splicing event (exons 7 and 8 skipping) is the most prevalent. Abbreviations: ACC: adrenocortical carcinoma; BLCA = bladder urothelial carcinoma; BRCA = breast invasive carcinoma; CESC = cervical squamous cell carcinoma and endocervical adenocarcinoma; CHOL = cholangiocarcinoma; COAD = colon adenocarcinoma; DLBC = lymphoid neoplasm diffuse large B-cell lymphoma; DN = dominant-negative; ESCA = esophageal carcinoma; FL = full length; GBM = glioblastoma multiforme; HNSC = head and neck squamous cell carcinoma; *hTERT* = human telomerase reverse transcriptase; KICH = kidney chromophobe; KIRC = kidney renal clear cell carcinoma; LAML = acute myeloid leukemia; LGG = brain lower grade glioma; LIHC = liver hepatocellular carcinoma; LUAD = lung adenocarcinoma; LUSC = lung squamous cell carcinoma; MESO = mesothelioma; NMD = nonsense-mediated decay; OV = ovarian serous; cystadenocarcinoma; PAAD = pancreatic adenocarcinoma; PCPG = pheochromocytoma and paraganglioma; PRAD = prostate adenocarcinoma; READ = rectum adenocarcinoma; SARC = sarcoma; SKCM = skin cutaneous melanoma; STAD = stomach adenocarcinoma; TSVdb = the cancer genome atlas splice variant database; THCA = thyroid carcinoma; THYM = thymoma; UCEC = uterine corpus endometrial carcinoma; UCS: uterine carcinosarcoma.

To determine whether or not alternative splicing events within the exons 5–9 region of *hTERT* are in fact the most abundant in human cancers, we used publicly available RNA-sequencing data from The Cancer Genome Atlas (TCGA) splice variant database (TSVdb), a web tool which enables the acquisition of *hTERT* splice variant abundance across 33 tumors of which 31 cancer types had data available for *hTERT* [74]. The TSVdb RNA-sequencing data demonstrates that ~80.4% of available tumor samples are positive for *hTERT* transcripts, results which are consistent with previous reports [75]. Furthermore, the percentage of *hTERT* transcripts quantified by the TSVdb as FL averages ~22% and ranges from ~1% to ~54% across all tumor samples (Figure 1). Five additional alternatively spliced variants of *hTERT* were identified to contain one or more combination of the -α and -β deletions, and the -γ deletion that skips exon 11 within the RT-domain and may act as a dominant-negative inhibitor of telomerase in cancer cells [76] (Figure 1A). However, the percentage of alternatively spliced variants that would be detected by the exons 5–9 assay first used by Killian et al. [62] averages ~5% (1–11%) for -α, ~62% (40–79%) for -β, and ~6% (0–46%) for -α-β (Figure 1B). These data highlight the importance of *hTERT* RT-domain alternative splicing and that the -β variant (exon 7/8 skipping), is the most common event. Thus, understanding the regulation of this region is critically important to elucidating telomerase regulation as the splicing mechanisms involved might also be amenable to manipulation.

It is important to note that several limitations exist in measuring *hTERT.* For example, the gel-based exons 5–9 assay remains limited as a qualitative approach, valid only for the detection, but not quantification, of *hTERT* transcripts that may result in bias of shorter PCR amplicons. RNA-sequencing data are quantitative and capable of providing an absolute, and therefore, more accurate assessment of presence and proportion of *hTERT* transcripts present within a particular sample. However, RNA-sequencing is limited in its capacity to quantify *hTERT* transcript isoforms due to library preparation issues in capturing low abundant transcripts [77]. Further impeding accurate detection of *hTERT* transcript isoforms is the ‘GC’ content of the 5′ end of the *hTERT* gene (>70% in exons 1 and 2), which precludes detection using standard RT and PCR conditions, and thus limits the absolute quantification of each variant from RNA-sequencing analysis [78]. Therefore, the development of additional methodological approaches, such as long-read sequencing using third-generation technologies and capture/enrichment strategies, are necessary to enhance the ability to identify and quantify *hTERT* splicing variant isoforms for the accurate interpretation of patient data. For instance, Sayed et al. used Pacific Biosciences long-read sequencing with a targeted library preparation approach to point out differences in *TERT* splicing between mice and men [73]. Moving forward, additional steps could be taken to more carefully document the alternative splicing profile of *hTERT,* such as employing tiled probes along the span of *hTERT* exons and introns to enrich cDNA libraries for *hTERT* products that would allow for cataloging of *hTERT* isoforms with long-read sequencing technologies (e.g., Oxford Nanopore or Pacific Biosciences) [79]. An alternative approach, and one which avoids cDNA and PCR bias, involves the direct enrichment of RNA via pulldown of *hTERT* through the use of tiling probes followed by direct long-read RNA-sequencing [80]. Beyond the difficulty of accurately detecting *hTERT* alternative splicing, our laboratory and others have begun to dissect the regulatory mechanisms of *hTERT,* thus leading the way in the development of potential therapeutic approaches for targeting telomerase. 

## 3. Targeting Alternative RNA Splicing of *hTERT* as a Cancer Therapy 

Alternative RNA splicing of *hTERT* in cancer suggests a need for malignant cells to tightly regulate and produce the “right amount” of telomerase to maintain telomere lengths, but not to elongate telomere lengths so as to confer a negative impact on cellular survival (e.g., decreased tolerance to ionizing radiation) [81,82]. As such, mechanistically understanding *hTERT* alternative splicing of the -β variant offers the possibility of developing novel anticancer agents that reduce telomerase activity by shunting transcripts from the FL *hTERT* telomerase activity coding isoform to catalytically dead variants and attenuate cancer growth and survival by shortening telomeres. Although *hTERT* gene expression has been known to be regulated by alternative RNA splicing for nearly 25 years [34], the role of alternative RNA splicing in regulating *hTERT* as a potential therapeutic target during cellular development and in cancer has only recently been investigated [64,83]. The advancement of oligonucleotide chemistries and delivery modalities over the past 20 years makes the field primed to target telomerase by generating specific antisense oligonucleotides (ASO) aimed at the inhibition of telomerase. This is largely driven by recent FDA approvals of ASOs such as Nusinersen, leading to a resurgence in the interest in inhibiting telomerase via direct manipulation of *hTERT* [84]. To effectively target *hTERT* with ASO technologies, a deeper biochemical understanding of the molecular mechanisms, specifically the *cis*- and *trans*-acting factors, involved in the regulation of alternative RNA splicing is required. 

### 3.1. Cis-Elements Regulate hTERT Alternative RNA Splicing

**Figure 2:**
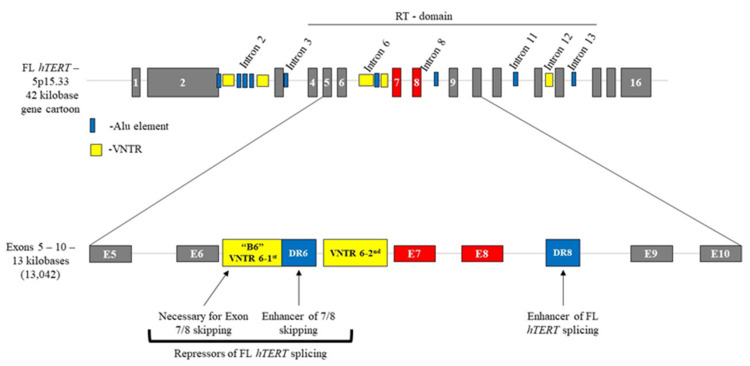
Known *cis*-elements within the *hTERT* gene/pre-mRNA. A cartoon demonstrating that there are nine known ALU sequences and five VNTR regions located within the introns of *hTERT.* Within the RT-domain of *hTERT,* block 6 (B6; located in intron 6) enhances skipping of exons 7 and 8 (-β), thereby repressing expression of the FL *hTERT* isoform. In addition, direct repeat 6 (DR6; located directly downstream of B6 in intron 6) augments the functional role of B6 to promote skipping of exons 7 and 8, and together, yield a catalytically inactive *hTERT* variant and telomerase negative cell phenotype that contributes to the progressive shortening of telomere lengths. On the contrary, direct repeat 8 (DR8; located within intron 8) acts as a known promoter of FL *hTERT* transcript necessary for catalytically active telomerase enzyme and telomere length maintenance. Abbreviations: B6 = block 6; DR6 = direct repeat 6; DR8 = direct repeat 8; FL = full length; *hTERT* = human telomerase reverse transcriptase; RT = reverse transcriptase; VNTR = variable number tandem repeat.

Initial attempts to understand the mechanistic machinery involved in the regulation of *hTERT* alternative splicing used a minigene construct spanning exons 5–10 [85]. Wong and colleagues identified deep intronic sequences, including a block of 26 short repeats of 38 nt sequences located in intron 6, termed “block 6 repeats” (B6, variable nucleotide polymorphism 6.1; Figure 2). Immediately following B6 was a 256 nt direct repeat in intron 6, termed direct repeat 6 (DR6, part of a larger ALUsq element), which shared 85% sequence homology with an additional 258 nt direct repeat located in intron 8, termed direct repeat 8 (DR8, part of a larger ALUsq element) [85]. The B6, DR6, and DR8 elements were identified to be conserved among old-world primates, including humans, and are absent in new-world monkeys and other mammals, including rodents [85,86]. Furthermore, their inclusion within the minigene was found to be necessary to recapitulate the endogenous splicing pattern of *hTERT* [85]. 

Follow-up minigene experiments containing the B6, DR6, and DR8 intronic sequences demonstrate that removing B6 from the minigene construct fails to produce significant amounts of the -β variant, whereas B6 alone (DR6 and DR8 removed) results in the enhanced skipping of exons 7 and 8 and the increased expression of the -β variant compared to the “full” minigene (Figure 2). While DR6 was shown to help augment the capacity of B6 to produce the -β variant and reduce FL production, DR8 was shown to be necessary for the production of FL *hTERT* [85]. In a subsequent study, Wong et al. further demonstrated that B6 undergoes RNA:RNA pairing with complementary sequences within intron 8, thereby bringing the 5′ SS of exon 6 closer to the 3′ SS of exon 9 to promote skipping of exons 7 and 8 and the formation of the -β variant [86]. These findings highlight the functional role of each of the three deep intronic *cis*-elements, and their potential for intramolecular interactions in the tight regulation of FL *hTERT* and the production of the alternatively spliced -β variant. Results from these investigations further indicate that *hTERT* may not follow the canonical rules involved in the regulation of alternative splicing. Likewise, they were the first to demonstrate that the intronic *cis*-elements B6, DR6, and DR8 may have recently evolved as an additional regulatory region to control the abundance of FL *hTERT* via post-transcriptional alternative RNA splicing to “fine-tune” telomerase enzyme activity, tightly regulate telomere lengths, and limit cellular replicative capacity as an advanced tumor suppressor mechanism in large, long-lived mammals [21,87]. 

**Figure 3:**
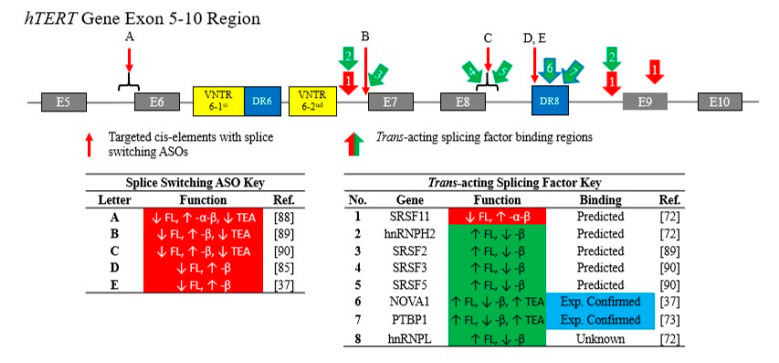
Therapeutically targeting *hTERT* splicing. Five regions within the RT-domain of *hTERT* pre-mRNA have been identified as potential *cis*-elements that are targetable with splice switching ASOs. Each has been identified to promote the alternative splicing of *hTERT* away from the FL transcript towards either the -β or -α-β variants and decrease telomerase enzyme activity in cancer cells (red arrows noted by the letters “A-E”). In addition, eight *trans*-acting RNA binding proteins have been identified to regulate *hTERT* FL splicing, either by repressing (red arrows noted by the number “1”) or enhancing the inclusion of exons 7 and 8 (green arrows noted by the numbers “2–8”). Although the majority of *trans*-acting splicing factors have been predicted to bind within the RT-domain, only NOVA1 and PTBP1 have been experimentally confirmed to directly bind to *hTERT* pre-mRNA within the DR8 region and promote the FL *hTERT*, telomerase activity, and telomere length maintenance in cancer cells. Abbreviations: ASO = antisense oligonucleotide; B6 = block 6; DR6 = direct repeat 6; DR8 = direct repeat 8; hnRNP = heterogeneous nuclear ribonucleoproteins; *hTERT* = human telomerase reverse transcriptase; PTBP1 = polypyrimidine-tract binding protein 1; RT = reverse transcriptase; SF = splicing factor; SRSF = serine/arginine-rich splicing factor; TEA = telomerase enzyme activity; VNTR = variable number tandem repeat.

Attempts to further understand the functional role of these *cis*-elements and their capacity to regulate the production of the FL *hTERT* and -β variants were initiated by the use of an antisense 2-*O*-methyl oligonucleotide against DR8 (Figure 3). As a result, a 20-mer beginning at the 19th-nt at the 5′ end of DR8 was sufficient to shift *hTERT* splicing from ~40% FL and ~50% -β to predominantly -β (<15% FL and >70% -β) [85]. Numerous studies have since investigated the use of ASOs to identify *cis*-elements and potential *trans*-acting RNA binding factors across *hTERT* pre-mRNA. Ludlow et al. targeted the endogenous *hTERT* gene within two non-small cell lung cancer (NSCLC) lines – H1299 and Calu6 – using a 2’-deoxy-2’-fluoro-arabinonucleic acid ASO that recognizes the same sequence within DR8 as Wong et al. [37,85]. Interestingly, transfection of H1299 with ASOs directed at DR8 produced a similar shift in *hTERT* splicing from the FL to -β variant, whereas no changes were observed in the Calu6 line [37]. These findings highlight that although the deep intronic *cis*-element DR8 may be involved in the recruitment of or act as a binding site for *trans*-acting factors that regulate *hTERT* splicing, the capacity to manipulate splicing choice through DR8 occurs is cell type specific. Thus, the use of splice switching ASOs on various cell lines of a particular tumor type supports the notion that the splicing code is cell specific and may necessitate the development of cell specific therapeutic strategies and/or the identification of a more universal *cis*-element to target with ASOs. 

It is likely that additional regulatory sequences within *hTERT* have yet to be identified and thus more research is needed to determine if a universal element exists controlling *hTERT* splicing. Indeed, other groups have attempted to use splice switching ASOs beyond DR8 to switch the splicing of *hTERT* towards either catalytically dead, dominant-negative isoforms or isoforms that undergo nonsense-mediated decay (Figure 3). For instance, Brambilla et al. used 2’-*O*-methyl antisense oligonucleotides targeting the intron 5/exon 6 boundary that resulted in a splicing switch from FL *hTERT* towards the -α-β variant, reduced telomerase activity, and generated a telomere length independent induction of apoptosis in the DU145 prostate cancer cell line [88] (Figure 3). While additional research is needed to confirm these results, it was the first demonstration that splice switching of *hTERT* with oligonucleotides is possible. More recently, two groups have also attempted to switch the splicing of *hTERT* by targeting intronic sequences. For example, Wang et al. targeted a region at the 3′ end of intron 6 containing predicted SRSF2 binding motifs with 2’-*O*-methyl splice switching ASOs that reduced *hTERT* FL, increased -β variant expression, and decreased telomerase enzyme activity in various brain cancer cells [89]. In addition, Zhdanov et al. targeted the exon 8/intron 8 junction at a region predicted to contain SRSF3 and SRSF5 binding sites and demonstrated reductions in FL *hTERT* splicing, increased -β production, and reduced telomerase activity in human immune cells [90]. Overall, these data indicate that targeting *hTERT* alternative splicing is possible with splice switching ASO technologies. Moving forward, more research into additional *cis*-elements and optimization of splice switching ASO targeting sequences, chemistries, and delivery will need to be pursued before important pre-clinical studies can take place. Understanding the potentially novel role these deep intronic ALU and variable number tandem repeats sequences play in the regulation of *hTERT* will not only inform the telomerase field, but also the roles these elements more broadly play in gene regulation.

### 3.2. Trans-Acting RNA-Binding Proteins Regulate hTERT Alternative RNA Splicing

Listerman et al. identified three potential *trans*-acting RNA binding proteins predicted to bind to introns 6 and 8 of *hTERT* pre-mRNA and regulate the production of FL and -β splice variants in various breast cancer cell lines [72]. Using a splicing reporter minigene consisting of *hTERT* exons 5 through 9, Listerman and colleagues demonstrated that the over expression of SRSF11 strongly promoted a shift in splicing from FL to the -β *hTERT* variant [72]. SRSF11 was then predicted to bind upstream of the 3′ SS within intron 6 and intron 8, as well as a potential site within exon 9. On the contrary, overexpression of hnRNPL and hnRNPH2 decreased -β production in favor FL *hTERT*, with hnRNPH2 being predicted to bind sites that overlapped those of SRSF11. Thus, it is likely that potential *trans*-acting RNA-binding proteins, such as SRSF11 and hnRNPH2, compete for binding within important *cis*-elements to dictate a cell’s splicing fate. While these findings also provide insight into potential therapeutic strategies to regulate *hTERT* splicing, and thus telomerase activity and telomere lengths, it is important to note that Listerman et al. also observed that overexpression of the -β variant protects breast cancer cells from apoptotic death [72]. As such, it may be that if the alternatively spliced -β variant reaches high expression levels (such as those common with overexpression constructs), it will allow some -β transcripts to escape nonsense-mediated decay and to generate a protein with novel functions.

More recently, Ludlow et al. used a HeLa cell minigene construct to perform a dual-luciferase small interfering RNA (siRNA) screen to determine the impact of 516 *trans*-acting RNA binding proteins of *hTERT* FL splicing choice [37]. Transient knockdown of 17 genes resulted in a two-fold increase in the production of the FL variant, whereas the knockdown of 93 genes resulted in a two-fold shift increase in the production of the -β variant. Ludlow and colleagues then applied a bioinformatics approach to narrow down the potential 93 genes involved in the promotion of *hTERT* FL in non-small cell lung cancer cells—identifying NOVA1 as a lead candidate for a therapeutic target in telomerase-positive cancers [37].

NOVA1 is an RNA-binding protein important for neuronal development [91]. Although NOVA1 is not expressed in most human adult tissues such as normal human bronchial epithelial cells, over 70% of lung cancers cell lines have been shown to express NOVA1 [37,73]. NOVA1 binds to clustered YCAY motifs, where Y equals either a C or T/U. There are five YCAY motifs located within DR6 and seven located within DR8. In subsequent experiments, Ludlow et al. used ultraviolet-crosslinking and immunoprecipitation techniques to demonstrate that NOVA1 binding is enriched at the 5′ end of DR8 and promotes *hTERT* FL splicing in the H1299 NSCLC line [37]. Therefore, the presence of NOVA1 at DR8 in NSCLC increases telomerase enzyme activity to maintain telomere lengths as a mechanism to sustain the growth and survival in vitro. However, the targeted deletion of DR8 or mutations (YCAY to YAAY) to the NOVA1 binding motifs using CRISPR/Cas-9 in H1299 cells prevented the interactions of NOVA1 with the DR8 region of *hTERT* pre-mRNA, thereby shifting *hTERT* splicing towards production of the -β variant, decreasing telomerase enzyme activity, and progressively shortening telomere lengths in cells [37]. These findings were the first to identify NOVA1 as a *trans*-acting RNA binding protein that directly binds to the DR8 region of *hTERT* pre-mRNA to promote cancer cell immortality. 

In a follow-up investigation, Sayed et al. identified that polypyrimidine tracts are located near the cluster of YCAY binding motifs within the DR8 region of *hTERT* pre-mRNA [73]. Polypyrimidine tracts are regions of pre-mRNA that promote spliceosome assembly and act as binding motifs for splicing regulators, including the polypyrimidine-tract binding protein 1 (PTBP1). PTBP1 is ubiquitously expressed in tissues and is overexpressed across cancer cell lines, regardless of NOVA1 expression patterns [73]. Similar to NOVA1, Sayed et al. demonstrated that PTBP1 knockdown shifts *hTERT* splicing from FL primarily to -β, decreases telomerase, and progressively shortens telomere lengths in the NOVA1 positive NSCLC lines H1299 and H920 in vitro [73]. Interestingly, although there are no telomere-related effects of PTBP1 knockdown on the NOVA1-negative NSCLC line Calu6, overexpression of NOVA1 in Calu6 cells restores the capacity of PTBP1 knockdown to shift *hTERT* splicing towards the -β variant and reduce telomerase enzyme activity. Using RNA pulldown assays, Sayed et al. further demonstrated that NOVA1 is both necessary and sufficient to recruit PTBP1 to DR8 and directs its binding directly downstream of the NOVA1 YCAY binding motifs. These observations indicate a NOVA1-PTBP1 splicing network that acts to promote FL *hTERT,* increase telomerase enzyme activity, and maintain telomere lengths in NSCLC [73]. These findings, together with previously identified *trans*-acting RNA-binding factors (Figure 3), have provided a working model that *hTERT* splicing enables cancer cells to become immortal through a yet to be determined splicing code.

## 4. Perspectives 

**Figure 4:**
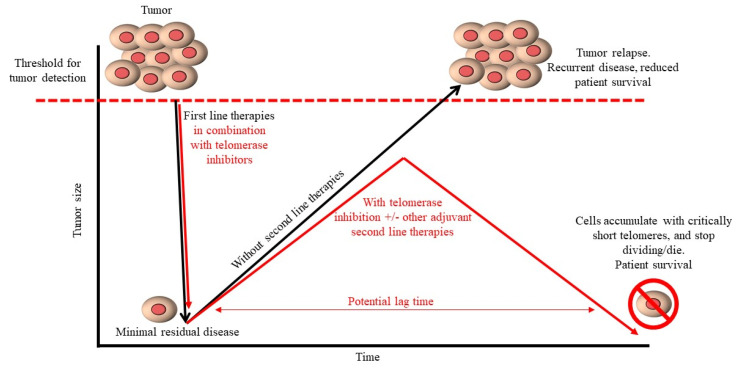
Potential use of telomerase inhibitors as cancer therapeutics. First line-therapies (surgery, radiation, and chemotherapy) aim to minimize residue levels of cancerous cells. However, there is risk associated with residual cancerous cells remaining viable and possessing the capacity for continued replication due to various mechanisms, including retaining telomerase activity and telomere length maintenance mechanisms. As a result, tumors may reoccur and reduce patient survival rates. The use of telomerase inhibitors simultaneously with first line therapies or as a second line therapy may help to prevent regrowth of remaining tumor cells by shortening telomeres to critically shortened lengths overtime. “Lag time” refers to the time (i.e., cell divisions) it takes for a telomerase inhibitor to shorten telomeres, reduce cell fitness, and prevent cancer growth. Such a strategy would help to reduce the capacity of cancerous cells to divide and promote cell death via apoptosis, thereby helping to increase patient survival.

Cancer is a major public health concern and understanding the regulation of *hTERT* alternative RNA splicing is important as a strategy to help prevent and treat telomerase positive cancers. For example, the prophylactic delivery of molecules targeting the *cis*-elements and/or *trans*-acting factors to prevent FL *hTERT* splicing and telomerase activity may help minimize cancer developments. We propose the use of a telomerase inhibitor as a cancer prevention strategy would only be indicated in populations with a genetic predisposition to cancer or a history of chronic exposure to cancer causing environmental stressors (e.g., smoking tobacco), by limiting the number of cells that develop immortality. In the event of cancer diagnosis, ASOs aimed at switching the splicing of *hTERT* could be given concurrently as a telomerase inhibitor while patients receive first line therapies (surgery, radiation, and chemotherapies). In addition, delivery of similar anti-telomerase molecules as an adjuvant along with anti-metastatic therapeutics aimed at preventing tumor survival may act additively and/or synergistically. Such an approach would enable the application of primary anticancer therapies targeting cancer growth and survival while simultaneously providing an opportunity to shorten telomere lengths of cancerous cells. Similarly, telomerase inhibitors, *hTERT* splice inhibitors included, can be continued as a second line therapy to help prevent regrowth of remaining tumor cells during remission, thereby increasing rates of patient survival compared with first-line therapies alone [92] (Figure 4). Further applying precision medicine approaches could help to identify tumors with short telomeres as stronger candidates for telomerase inhibitors to limit/reduce the lag time associated with telomere length shortening and cancer cell death compared to tumors with longer telomeres [92,93]. Screening tumors for *hTERT* isoform ratios may also provide additional insights into the types of anti-telomerase therapies that could potentially be applied. Therefore, continued efforts are necessary to gain a more complete understanding of regulatory mechanisms involved in *hTERT* alternative RNA splicing regulation as a potential approach to developing novel anticancer therapies. 

## 5. Conclusions 

Telomerase is a near universal target for cancer therapeutic strategies. Although several strategies for targeting telomerase positive cancers have been attempted over the past 25 years, few have progressed clinically due to issues related to the length of time and number of cell divisions needed to drive cells into telomere-induced crisis (i.e., lag times), poor in vivo inhibition of the enzyme, and potential off target toxicities, including reduced platelet counts [94]. Therefore, the identification of new targets and the application of new therapeutic approaches are required to deactivate the telomere maintenance mechanisms involved in cancer growth and survival. The transcriptional and post-transcriptional regulation of the *hTERT* gene have gained increased attention in recent years. By continuing to increase focus on the identification of potential *cis*-elements and the network of *trans*-acting factors that comprise the *hTERT* splicing code, additional therapeutic opportunities are likely to emerge. The idea of switching the splicing of *hTERT* from telomerase encoding FL to alternative spliced catalytically inactive isoforms is attractive for inhibiting telomerase and shortening telomeres in cancer cells. For this to become reality, a deeper understanding of the *cis*- and *trans*-acting factors must be achieved similar to what has been achieved by scientists studying spinal motor neuron 1 and 2 or dystrophin genes [95,96].
